# Little Known Facts, Controversies and Misconceptions About Cranial Motor Nuclei (and a New Classification for All Motoneurons, Cranial, and Spinal)

**DOI:** 10.1002/cne.70153

**Published:** 2026-04-14

**Authors:** Margaux Sivori, Bowen Dempsey, Jean‐François Brunet

**Affiliations:** ^1^ Institut de Biologie de l'ENS (IBENS), INSERM, CNRS, École Normale Supérieure PSL Research University Paris France; ^2^ Faculty of Medicine, Health & Human Sciences Macquarie University Macquarie Park New South Wales Australia

## Abstract

Cranial nerves represent a notoriously complex province of the neuroanatomical landscape of the vertebrates. Here, we offer a selection of the anatomic, genetic, and developmental features of their efferent component that are often misrepresented, ignored or controversial, as a complement to more exhaustive treatments of the subject. Our description reveals that efferent (or “motor”) neurons in vertebrates represent a vague anatomic category (such as that of interneurons) rather than a true neuron type; That motor neurons fall into three bona fide types, segregated on the rostro‐caudal axis of the central nervous system; That each of the three types is highly related to a type of preganglionic autonomic neuron; and that this genetic and topographical arrangement of three motor/preganglionic types correlates, not perfectly yet remarkably, with three broad physiological functions.

## Abstract 2

All efferent neurons of the vertebrate fall in three cardinal classes, developmentally distinct and segregated on the rostro‐caudal axis of the central nervous system, each class being divided into two genetically related subclasses: direct (classical motoneurons) and indirect (autonomic preganglionic).

## 1 Introduction

From the outset, the term “motoneuron,” concerning vertebrates, has carried an ambiguous meaning, for the reason that it was bestowed not only on neurons of the central nervous system that project in the periphery to synapse onto muscles, but also to “visceral (or splanchnic) motoneurons” most of which project onto autonomic ganglia, which cannot be said to be motorized; conversely the term was never commonly used for these autonomic ganglionic neurons, many of which have always been known to synapse onto muscles (e.g., cardiac, vascular, and digestive). This nomenclatural awkwardness likely arose from the ignorance of the true nature of autonomic ganglia as synaptic relays: for a long time, they were seen as mere enlargements, of uncertain function, on the trajectory of nerves *en route* to visceral muscles. In addition to “motoneuron,” another term has often been used to subsume conventional motoneurons and preganglionic neurons: that of “efferent” (which for example dominates the discourse of Gaskell (Gaskell 1886)). As we shall see in this review, “efferent” has a number of advantages over “motoneuron”: it dispels the appearance of a confusion between motoneurons in the most habitual sense and autonomic preganglionics (the conflation being legitimate, as we shall see); avoids the suggestion of false categories (that of conventional “motoneuron” in the first place, as we shall explain); and allows the introduction of real ones, based on developmental genetics. We shall thus abundantly make use of the term “efferent,” without unduly upsetting conventional terminology.

**FIGURE 1:**
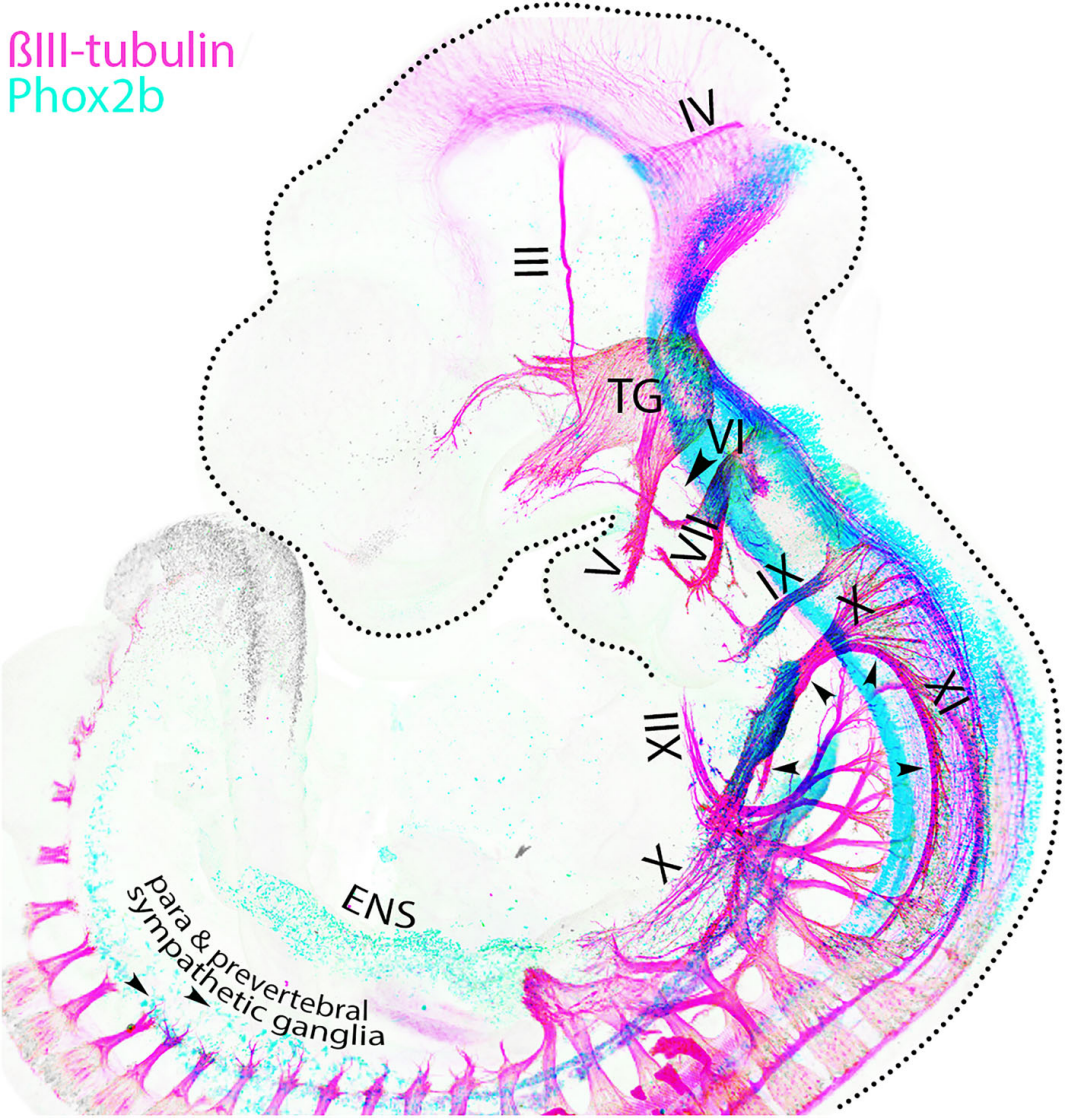
General arrangement of cranial efferents in a mouse embryo. Lateral view of a wholemount of a mouse embryo at gestational day 10.5 stained for the transcription factor Phox2b (that marks central and peripheral neurons of the visceral nervous system) and βIII‐tubulin (that marks all axons), showing the forming cranial efferent nerves, numbered in roman numerals. (III) oculomotor; (IV) trochlear; (V) mandibular branch of trigeminal nerve; (VI) abducens (with an arrowhead); (VII) facial; (IX) glossopharyngeal; (X) vagus; (XI) spinal “accessory”; (XII) hypoglossal. ENS: enteric nervous system; TG: trigeminal ganglion; the four small arrowheads indicate the trajectory of nXI (see Part 3.2).

**FIGURE 2:**
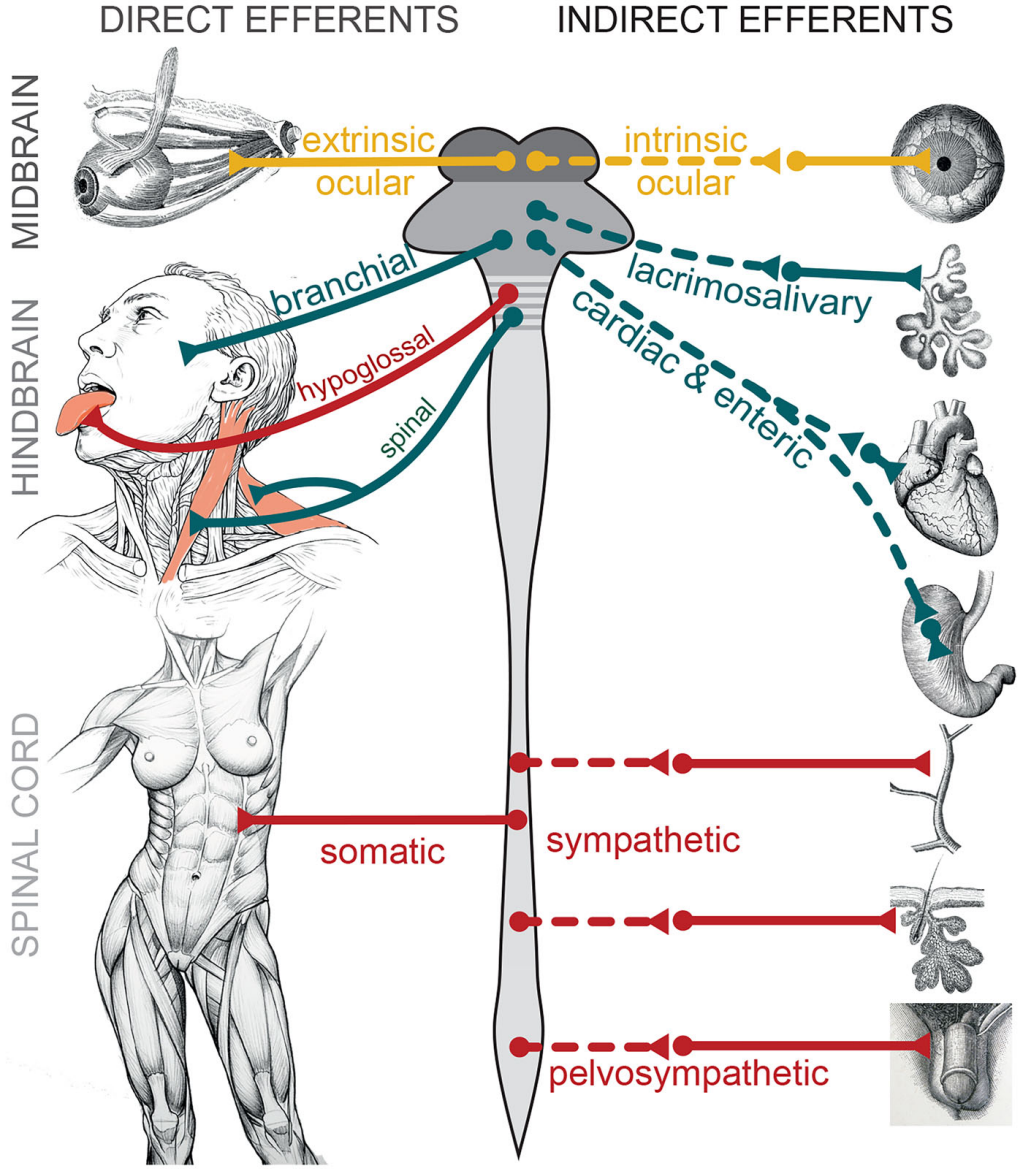
Classification defended in this review for efferents in the vertebrates, direct (left) and indirect (right). The midbrain gives rise to direct efferents for extrinsic ocular muscles, and indirect ones for intrinsic eye muscles via the ciliary ganglion (yellow). The hindbrain gives rise mostly to direct “branchial motoneurons” and indirect efferents to salivary glands, digestive tract, and heart (via dedicated ganglia) (blue). The spinal cord gives rise to “somatic motoneurons” for trunk muscles and indirect efferents for vascular, piloerector, and glandular targets, via sympathetic ganglia (red). The hindbrain contains two outliers of the spinal/somatic series of direct efferents (hypoglossal (Mo12) and abducens (Mo6), the latter not shown, see Table 1 and text for details); and the spinal cord contains one outlier of the hindbrain/branchiomotor series (spinal, Mo11). This marginal overlap in the distribution of efferent types is represented by the striped region of the central nervous system. Because of the kinship between direct and indirect efferents, the most convenient term to designate globally each class is by its location (midbrain, hindbrain, and spinal efferent), keeping in mind the topographical oddities of Mo6, Mo11, and Mo12.

**FIGURE 3:**
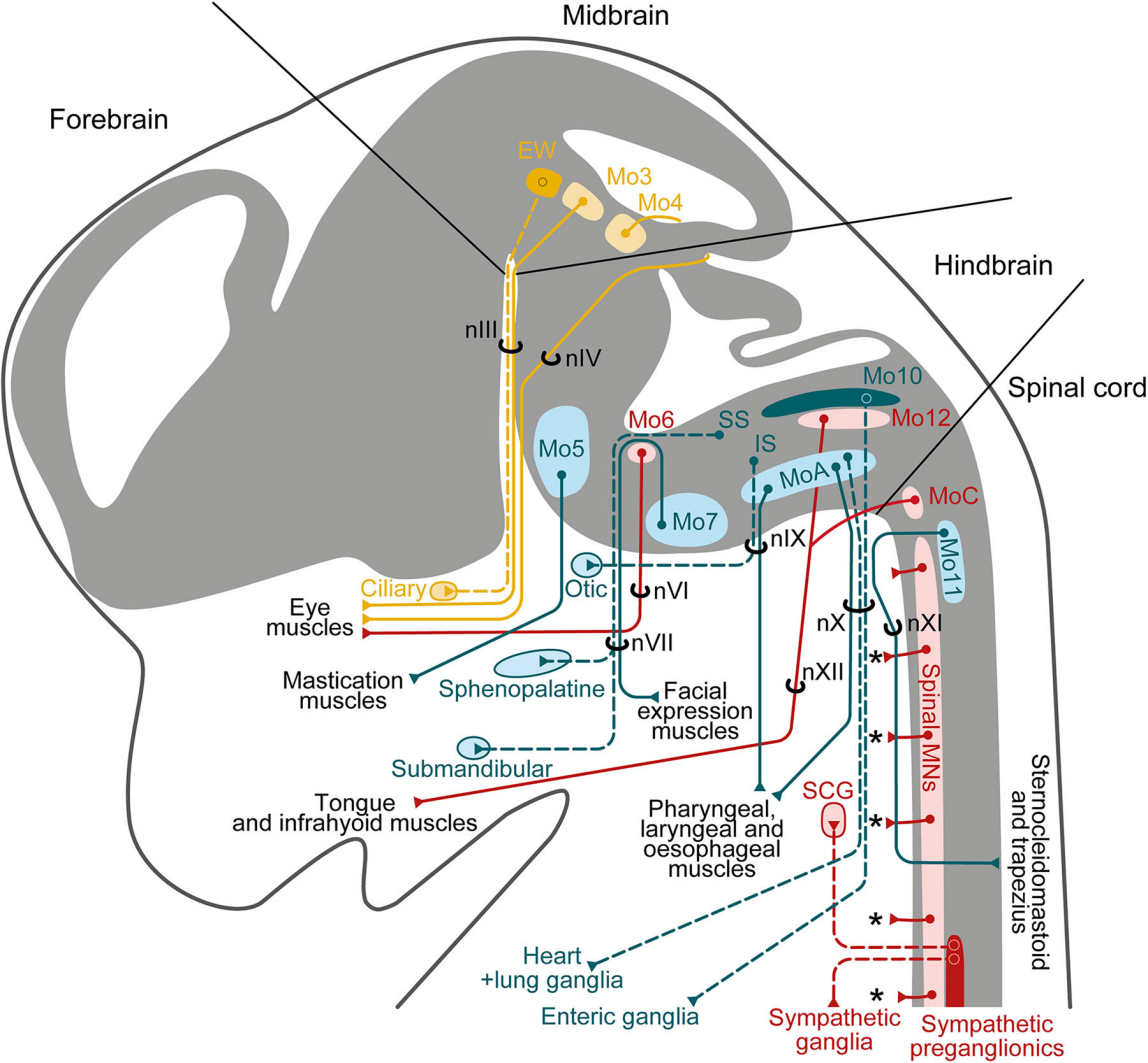
Semi‐schematic representation of cranial and cervical motor nerves in a mouse, classified according to Figure 2, Tables 1 and 2. Yellow: midbrain efferents; blue: hindbrain efferents; red: spinal efferents. Light colors: in the CNS, direct efferent nuclei; in the periphery, ganglionic relays; dark colors: indirect efferent nuclei. Solid lines: direct efferent nerves; stippled lines: indirect efferent nerves. EW: Edinger‐Westphal nucleus; IS: inferior salivary nucleus; Mo3‐12 and MoC: motor nuclei; nIII‐nXII: cranial nerves; SCG, superior cervical ganglion; SS: superior salivary nucleus; asterisks: limb and trunk muscles. The superior and inferior salivary “nuclei” are represented by simple dots because they do not form bona fide nuclei. Heart preganglionics of the hindbrain are found mostly inside and around MoA (Nosaka et al. 1979), (Hopkins and Armour 1982), (Stuesse 1982) (simplified on the schematic as: inside MoA), together with preganglionics for lung ganglia (Veerakumar et al. 2022), but also in Mo10 (Nosaka et al. 1979); (Kalia 1981) (not represented). IEEs are not represented.

Here we review the cranial nerves (Figure 1) by highlighting developmental features which are consistent with a novel classification of all efferents (including spinal) (Figures 2 and 3, Table 1) based on genetics (Table 2), into three cardinal classes: each occupy a different rostro‐caudal region of the central nervous system, each is dedicated to a different set of targets, and each is divided into two highly related subclasses: motoneurons in the strict sense (that we call “direct” [efferents or motoneurons] to denote that they directly synapse onto muscles), and preganglionic autonomic neurons (that we call “indirect” [efferents or motoneurons]) to denote that they also control muscles (and other targets), but via a ganglionic relay.

**TABLE 1:** List of efferents of the vertebrate classified according to the genetic criteria synthesized in this review (see Table 2). Direct efferents and their targets are in regular font, indirect efferents and their targets in italics. Color code (as in Figure 2 and Table 2): in the second column, yellow, blue, and red code for, respectively, the midbrain, hindbrain, and spinal classes of direct and indirect efferents and correspond to anatomic locations in the first column except for three outliers: two direct motor nuclei of the spinal class in the hindbrain and one direct motor nucleus of the hindbrain class in the cervical spinal cord.

Anatomic location	Nuclei and columns of direct and *indirect* efferents	Muscular and *ganglionic* targets
**Midbrain**	Mo3 Mo4 *Edinger‐Westphal*	Medial rectus, inferior rectus, superior rectus, inferior oblique of the eye Superior oblique of the eye *Ciliary ganglion*
**Hindbrain**	Mo5 Mo6 Mo7 MoAcc (5/7) IEE (Mo8) MoA *MoA external formation* *Mo10* *Salivatory “nuclei”* Mo12	The three jaw closers: masseter, pterygoid, and temporal Lateral rectus of the eye Muscles of the face (frontalis, zygomatic, orbicularis oris, platysma, etc.) The four suprahyoids (jaw openers): posterior and anterior digastric, stylohyoid, mylohyoid. Outer hair cells of the cochlea and sensory terminals in vestibule and cochlea Laryngeal, pharyngeal, and esophageal muscles. *Cardiac and pulmonary ganglia* (and cricothyroid muscle) *Enteric ganglia and cardiac ganglia* *Sphenopalatine, otic, submandibular, and lingual ganglia* 12 tongue muscles + geniohyoid
**Spinal cord**	MoC Mo11 MMC LMC *IML/DN*	The four infrahyoids: omohyoid, sternohyoid, sternothyroid, thyrohyoid Sternocleidomastoid, trapezoid Epaxial muscles/hypaxial muscles/diaphragm Limb muscles, dorsal/ventral *Sympathetic and pelvic ganglia, adrenal medulla*

Abbreviations: DN: dorsal nucleus; IEE: inner ear efferents (also called cochleovestibular efferents); IML: Intermediolateral column; LMC: Lateral motor column; MMC: Medial motor column; Mo: Motor nucleus.

**TABLE 2:** Transcriptional markers and determinants of the three cardinal classes of efferents with main bibliographic references. The three classes are grouped and color coded as elsewhere in the review and designated by the anatomic location of most of their representatives (midbrain, hindbrain, and spinal cord) even though two spinal‐type direct efferents (Mo6 and Mo12) are in the hindbrain and one hindbrain‐type direct efferent (Mo11) is in the spinal cord, (see Table 1 and main text). Markers or determinants shared between classes are coded in various shades of gray. The only developmental features common to all efferents and which could thus be construed as an element of "deep homology" (Shubin et al. 2009; Tschopp and Tabin 2017)—in this case, serial homology—are expression of *Islet* *1* and an origin in the basal plate (i.e. the ventral neural tube), more precisely a part or another, depending on classes, of the *Nkx6*‐expressing progenitor domain.

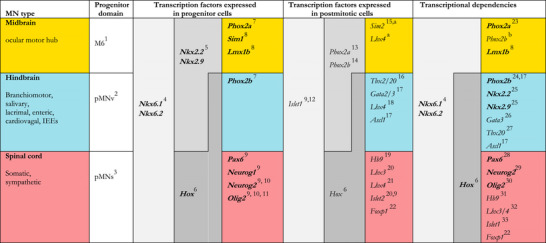

*Note:* Transcription factors expressed in progenitors are in bold italics, those that are expressed in post‐mitotic precursors in italics. Relevant references for the cranial outliers of the somatic series, Mo6 and Mo12 (often overlooked or buried in the small print), are provided explicitly when available. References showing that a marker is absent from a given class of neurons (e.g., *Phox2b* from somatic neurons, or *Hb9* from midbrain motoneurons) are not provided for the sake of manageability of the table and its legend. Cranial preganglionics have rarely been examined in terms of genetic dependencies, but their transcriptional make up is the same as for branchiomotor. **1** : (Nakatani et al. 2007) To our knowledge, this terminology has not been adopted beyond this reference and no standard nomenclature exists for dorsoventral progenitor domains of the midbrain; **2** : (Ericson et al. 1997; Jarrar, Vauti, et al. 2015); **3** : often simply called pMN, for example (Alaynick et al. 2011). **4** : Sander et al. (2000), Vallstedt et al. (2001) for spinal (including Mo6 and Mo12), Prakash et al. (2009) for Mo3, M. Müller et al. (2003) for hindbrain; **5** : Deng et al. (2011) for Mo3, Jarrar, Vauti, et al. (2015) for hindbrain efferents including Mo11, Kobayashi et al. (2013) for Mo11 ; **6** : Philippidou and Dasen (2013) for review ; **7** : (Pattyn et al. 1997), Kobayashi et al. (2013) for Mo11; **8** : (Deng et al. 2011), the effect of the *Lmx1b* KO on EW can be inferred from disappearance of all *Phox2a* cells; **9** : Alaynick et al. (2011) for review; **10** : Mizuguchi et al. (2001) for “somatic motoneurons of the hindbrain”; **11**: Novitch et al. (2001) for “unpublished data” for the caudal hindbrain, thus Mo12, Zannino and Appel (2009) for Mo6 in zebrafish; **12**: Varela‐Echavarría et al. (1996) for Mo6 and Mo12; **13** : (Tiveron et al. 1996) including for IEEs (VEN); **14** : Pattyn et al. (1997) for BM and Mo10, Kang et al. (2007) for BM, Mo10, salivary and IEEs (but only VEN and MOCs see Figure 9 for LOCs); **15**: only in indirect efferents (Caqueret et al. 2005). **16**: Dufour et al. (2006), Song et al. (2006); **17** : Tiveron et al. (2003) for IEEs ; **18** : (Gavalas et al. 2003; Kim et al. 2016), no data on indirect efferents ; **19** : (Alaynick et al. 2011) Rapidly extinguished in post‐mitotic indirect efferents according to William et al. (2003), but a *Hb9::GFP* transgene labels the *rami communicantes* to sympathetic ganglia Erickson et al. (2024), Tanabe et al. (1998), Guidato et al. (2003) for Mo6, Mo12; **20**: Tsuchida et al. (1994), Varela‐Echavarría et al. (1996) for Mo6, but claims only in accessory Mo6, Sharma et al. (1998) for Mo6, Mo12. **21** : Sharma et al. (1998) for spinal, Gavalas et al. (2003) for Mo6**; 22**: Dasen et al. (2008), Rousso et al. (2008) for spinal cord, no data on Mo6/Mo12; **23** : Nakano et al. (2001) for Mo3 in humans, (Hasan et al. 2010) by gain of function, including EW inferred from expression of *Sim2*, Pattyn et al. (1997) for all efferents including EW, inferred from disappearance of all peripherin signal, Guo et al. (1999) for Mo3 + Mo4 of fish; **24** : Pattyn, Hirsch, et al. (2000), Hirsch et al. (2007) for Mo11 ; **25** : Pabst et al. (2003) and Dillon et al. (2005) for Mo11 in *Nkx2.9* KO, Jarrar, Dias, et al. (2015) for all branchiomotor including Mo11 in double *Nkx2.2/2.9* KO, with a caudo–rostral gradient of penetrance, that leaves Mo5 largely intact,; **26** (Karis et al. 2001) specific to IEE; **27** : (Song et al. 2006), including IEEs and preganglionics ; **28** : Ericson et al. (1997) for Mo12 and spinal, Osumi et al. (1997) for Mo6 and Mo12, Mizuguchi et al. (2001) for “somatic motoneurons of the hindbrain” ; **29** : Scardigli et al. (2001) and Zannino and Appel (2009) for Mo6 in zebrafish. **30** : Rowitch et al. (2002), Lu et al. (2002) for the disappearance of *Hb9* ^+^ motoneurons in the hindbrain, Zhou and Anderson (2002) for Mo12 and sympathetics, Espinosa‐Medina et al. (2016) for sympathetics, Zannino and Appel (2009) for Mo6 in zebrafish ; **31**: (Thaler et al. 1999), including for Mo6; **32** : Sharma et al. (1998) reported the conversion of cervical somatic motoneurons into the nearby branchiomotor Mo11 by the double knockout of the post‐mitotic transcription factors *Lhx3/4*—somewhat surprisingly in retrospect: now that we know that the transcriptional codes of the spinal and branchial motor lineages diverge extensively at the level of dividing progenitors, it is unexpected that inactivation of *Lhx3/4* in the post‐mitotic progeny of one lineage would suffice to convert it into the progeny of the other. Moreover, *Lhx4* is shared by both neuron types (Gavalas et al. 2003; Kim et al. 2016). At least one evidence for the switch (the distribution of *ChAT*+ cells in *Lhx3/4* mutants [Figure 5c,d]), is actually compatible with the more likely ablation of the somatic motor column and preservation of the branchiomotor one. Further analysis of these mutants seems warranted. **33**: Pfaff et al. (1996) for spinal cord, Kim et al. (2016) for Mo12 and subtler phenotype for hindbrain efferents, no data on Mo6.

^a^ Sivori et al. *in preparation*; 2026

^b^ Postmitotic role in upregulation of *Sim2* (Sivori et al. 2026 *in preparation*).

The three classes are, from rostral to caudal: (i) midbrain efferents for the eye muscles, extrinsic, and intrinsic; (ii) hindbrain efferents for branchiomeric muscles (“branchiomotor” neurons), non‐sympathetic ganglia of the head and trunk, and hair cells of the inner ear; (iii) spinal efferents for somite‐derived muscles (“somatic” motoneurons) and for sympathetic ganglia. The latter two classes overlap at the fuzzy border between hindbrain and cervical spinal cord (Figure 2): the spinal cord harbors a caudal outlier of the hindbrain group and the hindbrain harbors rostral outliers of the spinal group (which justifies their treatment in a review on cranial efferents). We will use this classification to structure the review in three parts, and will let its justification emerge piecemeal.

We shall deliberately overlook two familiar ways of classifying direct efferents, on the ground that they most likely play out at lower hierarchical levels, that is *within* the great divisions that we propose. One is in motor *nuclei*, *columns*, and *pools* of direct efferents, each dedicated to a given muscle or muscle group: their genetic identity has been studied mostly in the spinal cord (e.g., Y. Lee et al. 2023; for reviews see (Jung and Dasen 2015; Shirasaki and Pfaff 2002) and references therein), more anecdotally in a hindbrain branchiomotor nucleus (Tenney et al. 2019) and not at all in the midbrain. This level of analysis has just begun for indirect (preganglionic) efferents of the spinal cord (Harima et al. 2024) or hindbrain (Tao et al. 2021; Veerakumar et al. 2022), revealing “paralleled labelled lines” of neural control for different autonomic targets. In the current state of knowledge, no data or rationale suggests that this parcellation could follow an overall, body‐wide logic. Another common categorization is according to the type of muscle fiber innervated by direct efferents: γ‐motoneurons for intrafusal fibers (in spindles) versus α motoneurons for extrafusal fibers; and motoneurons for “fast” versus “slow” motor units (Kanning et al. 2010). The extent to which the different types of muscle fibers themselves are identical at ocular, branchial, and trunk levels is uncertain, and at any rate, all available developmental and genetic data underlying this level of motoneuronal diversity concern the spinal cord (e.g., Alkaslasi et al. 2021; Ashrafi et al. 2012; Blum et al. 2021; Dasen et al. 2005; Enjin et al. 2010; Khan et al. 2022; D. Müller et al. 2014)^1^. Given the depth of genetic differences among the three cardinal classes that we summarize in Table 2, transcriptional signatures correlating, across them, with the type of muscle fiber innervated would represent a striking case of genetic convergence.

## 2 Midbrain Efferents

The first class of efferents reside in the caudal midbrain and mid‐hindbrain boundary (or isthmus)—both locations hereafter subsumed under the term “midbrain” for simplicity—and comprise, from caudal to rostral, the trochlear (Mo4), oculomotor (Mo3), and Edinger‐Westphal (EW) nuclei, all concerned with muscles of the eye, intrinsic and extrinsic (Figures 1, 2, 3 and Table 1). Collectively, Mo3 and Mo4 innervate five of the six *extrinsic* muscles of the eye: Mo4 innervates the superior oblique; Mo3 innervates the inferior oblique, the superior, medial, and inferior recti (as well as the levator palpebrae superioris). The sixth muscle, the lateral rectus, innervated by the “somatic” (i.e., spinal‐class) Mo6, constitutes an intriguing case of discordance between muscle type and nerve type that we shall examine in Part 4.1. Next to Mo3 lies the EW, which is preganglionic to the ciliary ganglion and, via this relay, contracts the *intrinsic* muscles of the eye: the ciliary muscle (for accommodation of the lens to distance) and pupillary sphincter (for accommodation of the pupil to light). The proximity of EW to Mo3 (indeed, the lack of clear cytoarchitectonic separation in many species), their common nerve of projection (the oculomotor nerve, nIII), and their common involvement in eye muscles, probably all played a role in inspiring the term “oculomotor complex” (OMC) for both nuclei together. A term is thus missing that would also include Mo4, highly related in ontogeny and function, despite its projection in a different nerve (nIV, the trochlear nerve). We propose the “ocular motor hub” (OMH) or, less exotically, the “midbrain efferents.”

### 2.1 Efferents for the Extrinsic Muscles of the Eye (Mo3 and Mo4)

**FIGURE 4:**
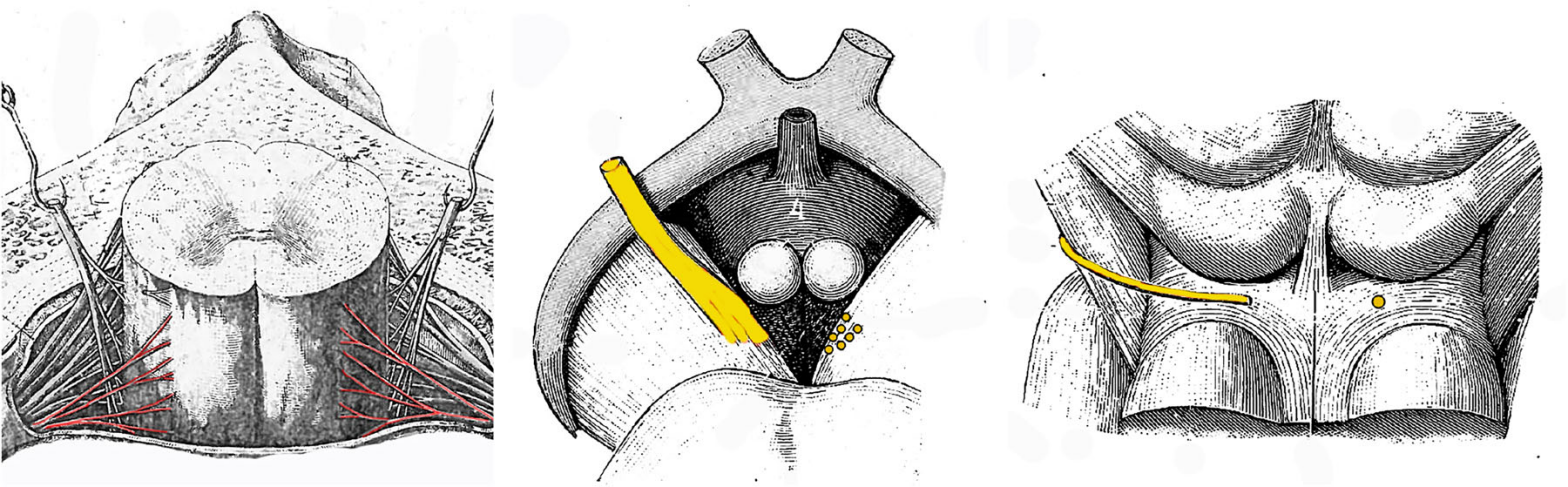
Contrasted modes of emergence of spinal and midbrain direct efferents. (**Left**) Ventrolateral roots of spinal motor nerves; (**center**), juxta‐medial roots of oculomotor nerve emerging from the inner face of the cerebral peduncle; (**right**) root of the trochlear nerve, on the dorsal surface of the isthmus (modified from Latarjet and Testut 1948). The three panels are not to scale.

An enduring misconception about Mo3 and Mo4 can be traced to Walter Holbrook Gaskell who sought to establish parallels between cranial and spinal nerves (Gaskell 1889)—a parallel that still permeates some contemporary textbooks, for example (Butler and Hodos 2005). His efforts themselves were probably rooted in the “transcendental anatomy” of the late 18th and early 19th centuries that promoted a fully metameric view of the vertebrate body, from tail to head. Thus, Gaskell put Mo3 and Mo4 in series with spinal somatic neurons. The rationale for a “somatic” label on Mo3 and Mo4 were twofold:

- They innervate striated muscles which were once thought to derive from head cavities or “head somites” detected in cartilaginous fishes (see Kuratani and Adachi 2016 and Noden and Francis‐West 2006 for historical accounts). Another, related reason might have been that the action on these muscles is voluntary (but this is not entirely true, and anyway this feature is shared by branchiomeric muscles).
- Mo3 has a ventral exit point, loosely reminiscent of the anterior root of spinal nerves. (But this is not true for Mo4, see below and Figure 4).

Gaskell's view was slightly amended by Herrick who, in the last version he gave of his “doctrine of nerve components” (Herrick 1918), classified Mo3 and Mo4 as “special somatic” (together with Mo6 and the hypoglossal nucleus, Mo12), ostensibly in relation to the “specialized” nature of the muscles that they innervate: the eye and tongue muscles. However this “special” nomenclature never stuck (see Fritzsch et al. 2017 for an exception), and Mo3/Mo4, have to this day most often been classified simply as “somatic” (e.g., Company et al. 2019; Gilland and Baker 2005; Hasan et al. 2010; Nieuwenhuys et al. 1998; Schubert et al. 2019; Watson et al. 2012) or even more explicitly, as “general somatic” (e.g., Butler and Hodos 2005; Tada and Kuratani 2015).

However, the reasons for this classification don't stand scrutiny any longer (and for some of them never did):

‐ *Concerning the target muscles:* The continuation of the somitic series in the head, in the form of “somitomeres” or “head cavities”, is now discounted (Noden and Francis‐West 2006) and the extraocular muscles are recognized as stemming from a dedicated mesodermal anlage and genetic program, distinct from those of both, somite‐derived and branchiomeric muscles (Sambasivan et al. 2009; Grimaldi and Tajbakhsh 2021).
‐ *Concerning the exit points of the nerves:* Those of Mo3 are actually more ventral than those of the spinal nerves, so that the vague notion of “ventral” confounds distinct topologies (Figure 4 left and center); and those of Mo4 trump this criterion entirely, since they are completely idiosyncratic: dorsal and contralateral, after a hidden course inside the mid‐hindbrain boundary (Figure 4 right).

All in all, little stands of the original arguments for a “somatic” (spinal‐like) classification—special or not—of Mo3 and Mo4. On the other hand, much has been learned that argues against it: the mode of formation of these two nuclei puts them in a “special” class indeed—like Herrick proposed—albeit unrelated to that of somatic motor neurons. Indeed, Mo3/4 differ from both, somatic (spinal) and branchial (hindbrain) direct efferents by the genetic code and dependencies of their progenitors (located in a specific progenitor domain of the midbrain, M6 (Nakatani et al. 2007)) and postmitotic precursors, listed in Table 2. If one compares the genetic make‐up of the progenitors of midbrain efferents with those of somatic and branchial ones, they have a slightly better claim at being “special branchial” than “special somatic”—on the account that their determinant, the paired‐like homeobox gene *Phox2a*, is the paralogue of *Phox2b*, the determinant of branchiomotor neurons of the hindbrain (which, however, poorly complements *Phox2a* in a knock‐in situation, including in Mo3 and Mo4 (Coppola et al. 2005)). Overall, they form a class by themselves.

### 2.2 Efferents for the Intrinsic Muscles of the Eye (EW)

Close to Mo3 (or within Mo3, depending on the species) lie the preganglionic neurons for the ciliary ganglion (Figure 3), first mentioned by Edinger (1885) and more decisively described by Westphal (1887), hence their name: they form the “Edinger‐Westphal nucleus”. Since (Loewy and Saper 1978) at least, the name was unfortunately bestowed on another nucleus, simply because of its proximity to Mo3 and its more conspicuous cytoarchitecture: this unrelated nucleus was basically confused for the EW^2^. The history of this mistake has been written (Kozicz et al. 2011). The term EW has become difficult to search in the recent literature, and the *Allen Brain Atlas* still uses the erroneous terminology (Burnell et al. 2008). Kozicz et al. (2011) proposed to mitigate the problem by the addition of a suffix—pg (“preganglionic”), for the nucleus described by Edinger and Westphal, and “cp” (“centrally projecting”), for the nearby unrelated nucleus, a solution rather unwieldy and possibly too accommodating^3^. A better one, which preserves the searchability of the second nucleus, while denoting the blunder from which its name arose and saving Edinger and Westphal from spinning in their graves, would be to call it the “pseudo‐Edinger‐Westphal” nucleus (pEW).

The original EW and its target, the ciliary ganglion, form the mesencephalic autonomic pathway to the eye (Figures 2 and 3). It was described by Gaskell as the “prosomatic outflow” (in a bold stroke of homologation with the arthropod's “prosoma” or anterior segments (Gaskell 1920, p.4). Later, it was integrated by Langley in his “parasympathetic” division of the autonomic nervous system, not without subtle misgivings: separating it from the “cranio‐sacral outflow,” even though it is cranial (Langley 1921, p.10) and remarking that it was “clearly distinct from the rest of the cranial outflow,” without explaining in what way (Langley 1921, p.8). And distinct it is, indeed, to the point that grouping EW together with hindbrain preganglionics creates a false category. By the criterion of all the gene expressions and dependencies that distinguish Mo3 and Mo4 from other direct efferents (see Table 2), the preganglionic neurons of EW resemble them, and differ from all other indirect (i.e., preganglionic) efferents. Indeed, they are the first example that we shall encounter of high ontogenetic relatedness between direct and indirect efferents at a given level of the neuraxis. Additional features, not encountered in other indirect efferents, are the expression of *Sim2* (Caqueret et al. 2005) and a postmitotic role for *Phox2b* in switching on *Sim2* (Sivori et al. 2026 in preparation). In line with the singularity of EW among indirect efferents, its target ganglion, the ciliary ganglion, represents a singular class of autonomic neurons (Sivori et al. 2026 in preparation).

All in all, the caudal part of the midbrain harbors a complex of efferent neurons, direct and indirect, highly related in their mode of development, as well as their physiological role in tuning the eye to the visual environment. The functional coordination of direct and indirect efferents is most dramatically exemplified in the “near triad,” that involves convergence (via Mo3), miosis and near accommodation (via EW) (Oyster 1999). Conversely, midbrain efferents are distinct, in their molecular make up, projection patterns and targets, from all other types of efferents, including the spinal direct (somatic) and hindbrain indirect (“parasympathetic”) types to which they have long been affiliated.

## 3 Hindbrain Efferents

Like the midbrain, the hindbrain—more precisely the portion that extends from the second rhombomere rostrally to an uncertain caudal border with the spinal cord caudally—harbors direct and indirect efferents (Figures 2 and 3, Tables 1 and 2). The direct ones are the “branchial motoneurons” (for muscles derived from the branchial arches), classically grouped in four nuclei: the trigeminal nucleus (Mo5), the facial nucleus (Mo7), the nucleus ambiguus (MoA) (which projects in both vagus and glossopharyngeal nerves, hence its “ambiguity” (Haines and Olry 1999)) and the so‐called “spinal accessory” nucleus (Mo11), actually located in the cervical spinal cord. They innervate branchiomeric muscles, derived from the branchial arch mesoderm, in a pattern that can be roughly summarized as: jaw closers and openers innervated by Mo5, additional jaw openers and facial expression muscles innervated by Mo7, pharyngeal, laryngeal, and esophageal muscles innervated by MoA and the sternocleidomastoid and trapezoid muscles innervated by Mo11. The indirect efferents are preganglionic to non‐sympathetic ganglia (classically called “parasympathetic” but see Brunet 2025) of the head and trunk: lacrimal, salivary, cardiac, pulmonary, and enteric. They form either loose cell assemblies—such as the improperly coined superior and inferior salivary “nuclei,” lateral and dorsal to Mo7 (Contreras et al. 1980) and the external formation of MoA—or bona fide nuclei such as the dorsal motor nucleus of the vagus nerve (Mo10), destined to enteric and cardiac ganglia (Nosaka et al. 1979). In addition to direct and indirect efferents, the myelencephalon contains a third subclass, the inner ear efferents (IEEs) that innervate the hair cells of the vestibule and cochlea. Finally, the hindbrain harbors two “somatic motor” nuclei, Mo6 and Mo12, which we treat as outliers of the spinal series, in Part 4.

**FIGURE 5:**
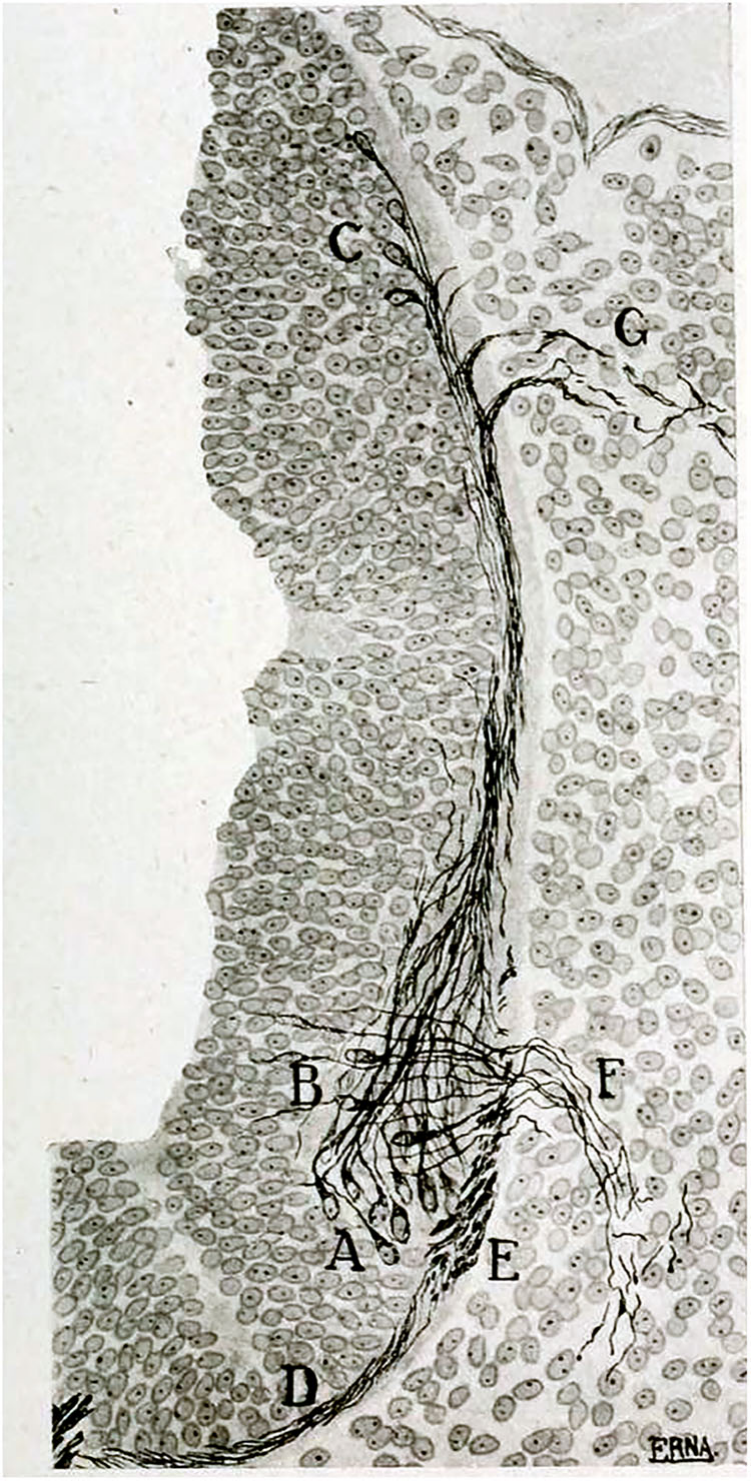
Formation of somatic and branchiomotor neurons in the caudal hindbrain as drawn in Figure 28 of (Tello, F. 1922): in a chick embryo at 70 h, precursors of the spinal "accessory" nucleus (A) whose axons have already reached a dorsal exit point (G), still lie close to their point of emergence, ventral to that of hypoglossal precursors (B) which project by a ventral root (F).

As was the situation in the midbrain, all hindbrain efferents (save Mo6 and Mo12), despite their diverse targets (branchiomeric muscles, autonomic ganglia, and hair cells) are highly related by their ontogeny and molecular profile. Conversely, they differ in many aspects from both midbrain and trunk ones. They arise from the ventral‐most neuroepithelial domain of the hindbrain and cervical spinal cord, dubbed pMNv (Ericson et al. 1997), adjacent to the more dorsal pMNs domain (that gives rise to somatic motoneurons). This is evident in the few places where they coexist: in rhombomere 5 (which in mammals gives rise to Mo6 and salivary preganglionics) and the caudal hindbrain/upper cervical spinal cord (which give rise to Mo12 + MoC (see Part 4) and the cranial and spinal roots of Mo11, see below)—the latter case suggestively documented as early as (Tello 1922) (Figure 5). Further caudally, the pMNv domain morphs into the p3 progenitor domain of the spinal cord, that gives rise to V3 interneurons (Briscoe et al. 1999). The transcription factor signature and transcriptional dependencies of hindbrain efferents are listed in Table 2.

We restrict our detailed treatment of hindbrain efferents to a particularity of Mo5 and Mo7 (their accessory nuclei), to controversial or misrepresented aspects of Mo11, and to the often‐overlooked IEEs.

### 3.1 The Accessory Trigeminal and Facial Nuclei (Acc5 and Acc7)

**FIGURE 6:**
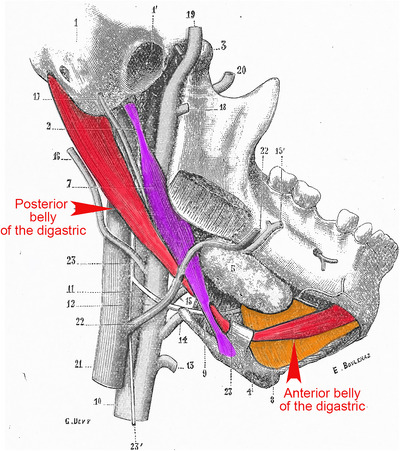
Anatomy of jaw openers. Anatomic drawing of the suprahyoid muscles (jaw openers) in humans, innervated by Acc5 (posterior belly of the digastric [red] and stylohyoid [mauve]) or Acc7 (anterior belly of the digastric [red] and mylohyoid [orange]) [modified from (Testut, L. 1911)].

The trigeminal motor nucleus (Mo5) and facial nucleus (Mo7) innervate the muscles of mastication and facial expression. Mo5 is often presented as a single nucleus, subdivided in a dorso‐lateral and a ventro‐medial division. A more realistic description (Paxinos and Franklin 2001) is to distinguish Mo5 proper (that innervates the jaw closers [temporal, masseter, and pterygoids]), and an “accessory nucleus” of Mo5 (Acc5) that innervates two jaw openers (the anterior digastric and mylohyoid). Similarly, Mo7, mainly destined to muscles of facial expression, is separated from an accessory nucleus, Acc7, that targets two additional jaw openers (the posterior digastric and stylohyoid) (Figure 6).

**FIGURE 7:**
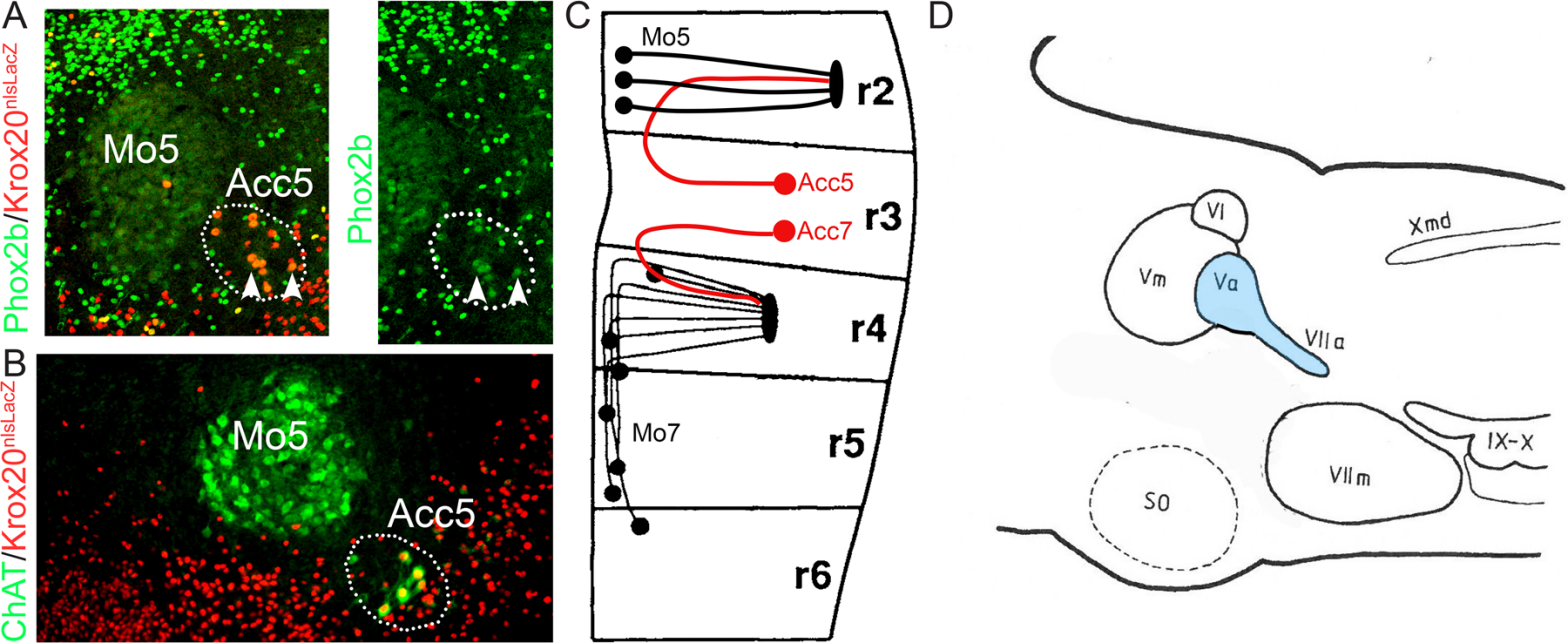
Developmental and anatomic affinities of Acc5 and Acc7. (**A**, **B**) Rhombomeric origin of Acc5. Coronal sections through the pons of *Krox20::Cre;nlsLacZ* embryos at P0, counterstained for Phox2b (A) or ChAT (B). All neurons of Acc5 (white arrowheads) express *nlsLacZ*; thus come from the territory of *Krox20* expression (in practice r3), while Mo5 comes almost exclusively from a *Krox20* negative territory (in practice r2). Note that *Phox2b* expression is weak in trigeminal motoneurons at this stage. (**C**) Summary of the origin of the trigeminal and facial nuclei, from our data and others, modified from Auclair et al. (1996) (who did not study Mo5 and missed the genu of Acc7). (**D**) Schematic of a parasagittal section of the hindbrain showing the accessory trigeminal and facial nuclei, in rat, as a single pear‐shaped nucleus (Va, VIIa), light blue (adapted from Székely and Matesz (1993), itself adapted from Szentágothai (1949)). The latter comments: “We may, therefore, say that the accessory facial nucleus is to be considered a caudal process of the trigeminal nucleus. The digastricus is therefore supplied by *a single column of cells*, the anterior part of which lies in the dorsoposterior part of the trigeminal nucleus (Author's note: now called the accessory trigeminal), the posterior forming the accessory facial nucleus. The roots for the anterior belly of the digastricus, from the anterior part of this column, join the trigeminus; the fibers originating from the posterior part of it, supplying the posterior belly, join the facial nerve. This may be the very simple explanation of the strange fact of innervation of the same muscle by two different nerves, which is extraordinary only among cranial nerves, but very common in the spinal cord, where fibers originating from the same cell column of the motor horn may emerge from the cord through different roots and may join again in the plexuses”. It is of note that, in humans, the anterior belly of the digastric, innervated by Acc5 (through the dental branch of nV), occasionally receives a branch from nVII (Kawai et al.^2003^). Whether the cells of origin are located ectopically in Acc7, or located in Acc5 and aberrantly project in nVII, this rare variant of innervation pattern is yet another illustration of the kinship of Acc5 and Acc7.

Distinguishing the main and accessory parts of both, facial and trigeminal nuclei, calls attention to a number of ontogenetic and anatomic features that set apart the two accessory nuclei from their respective main nucleus and, conversely, brings them together:

- *Their rhombomeric origin:* Developmentally, the dual rhombomeric origin of Mo5 (r2 and r3), first demonstrated in chicken (Lumsden and Keynes 1989), reflects in fish the partition between jaw adductors and abductors (Higashijima et al. 2000), and in mouse the separation of Mo5 and Acc5 (our data, Figure 7A–C). Conversely, the rhombomeric origin of Acc5, in r3, is close to that of Acc7, at the border between r3 and r4 (Auclair et al. 1996), prefiguring the proximity, in the adult, of Acc5 to Acc7 (see Matsuda et al. 1979 for guinea pig).
- *The morphology of their neuronal soma*, fusiform for both Acc5 and Acc7, as opposed to polygonal for Mo5 (Székely and Matesz 1982 in the rat).
- *The formation of a “genu”* by the axons from both Acc5 and Acc7 (like Mo7 but unlike Mo5) (in cat (Nomura and Mizuno 1983) and rat (Székely and Matesz 1982)) (Figure 7C), a testimony that the neuronal soma and growth cones migrated to their final lateral location independently of each other. Possibly related to this original migration pattern, both are slightly disorganized in *reeler* mutants, which leave Mo5 intact (Terashima et al. 1993; Terashima et al. 1994).
- *Last but not least, their function:* Acc5 and Acc7 synergize to mobilize supra‐hyoid jaw openers and antagonize Mo5, which innervates the mouth closers. They even share the innervation of a single muscle, the digastric, each being in charge of one of its two “bellies” (Figures 6 and 7).

For the reasons above, we can only stop short, for the sake of nomenclatural conservatism, of proposing that Acc7 and Acc5 constitute a single nucleus (MoAcc or Acc5/7) albeit dichotomous, with a trigeminal and a facial component, in reference to their nerves of projection. This view was in fact already proposed, as a “common nucleus of the trigeminal and facial nerve” (Székely and Matesz 1993) (Figure 7D) inspired by Szentágothai (1949).

### 3.2 Spinal “Accessory” Nucleus (Mo11)

The spinal accessory nucleus (SAN, better termed Mo11, see below) is located in the cervical spinal cord, gives rise to the “spinal accessory nerve” (better termed nXI, see below), and innervates two muscles in mammals, the sternocleidomastoid and trapezius (collectively homologous to the *cucullaris* of birds and fish).

The classification of Mo11 as a branchiomotor nucleus is ancient and classical (Herrick 1918; Watson et al. 2012), based on the dorsolateral exit point of its roots and the branchiomeric nature of the target muscles (e.g., Edgeworth 1935). In line with this view, Mo11 displays all the features that distinguish branchiomotor neurons from somatic ones (Hirsch et al. 2007; Kobayashi et al. 2013 Dillon et al. 2005; Jarrar, Dias, et al. 2015; Jarrar, Vauti, et al. 2015; Pabst et al. 2003) (see Table 2).

However, parallel to this well‐founded tradition, uncertainties and debates about nXI have kept running, from the ambiguous status of “accessory nerve” bestowed by Vieussens in 1684 (Swanson 2015) to recent placements of the nucleus and nerve in a class by themselves: “separate” and “unrelated to the vagus or branchial arch components” (Sperry and Boord 1993), “transitional” (Benninger and McNeil 2010), “intermediate/mixed” between somatic and branchial through some evolutionary trans‐differentiation from the former to the latter type (Tada and Kuratani 2015), “atypical” or “special” (Watson and Tvrdik 2019), or even in an “occipital group” together with the somatic Mo12 (Striedter and Northcutt 2019)! This marginal but undying hesitation might stem from two factors, one related to the nerve, the other to the muscles innervated.

**FIGURE 8:**
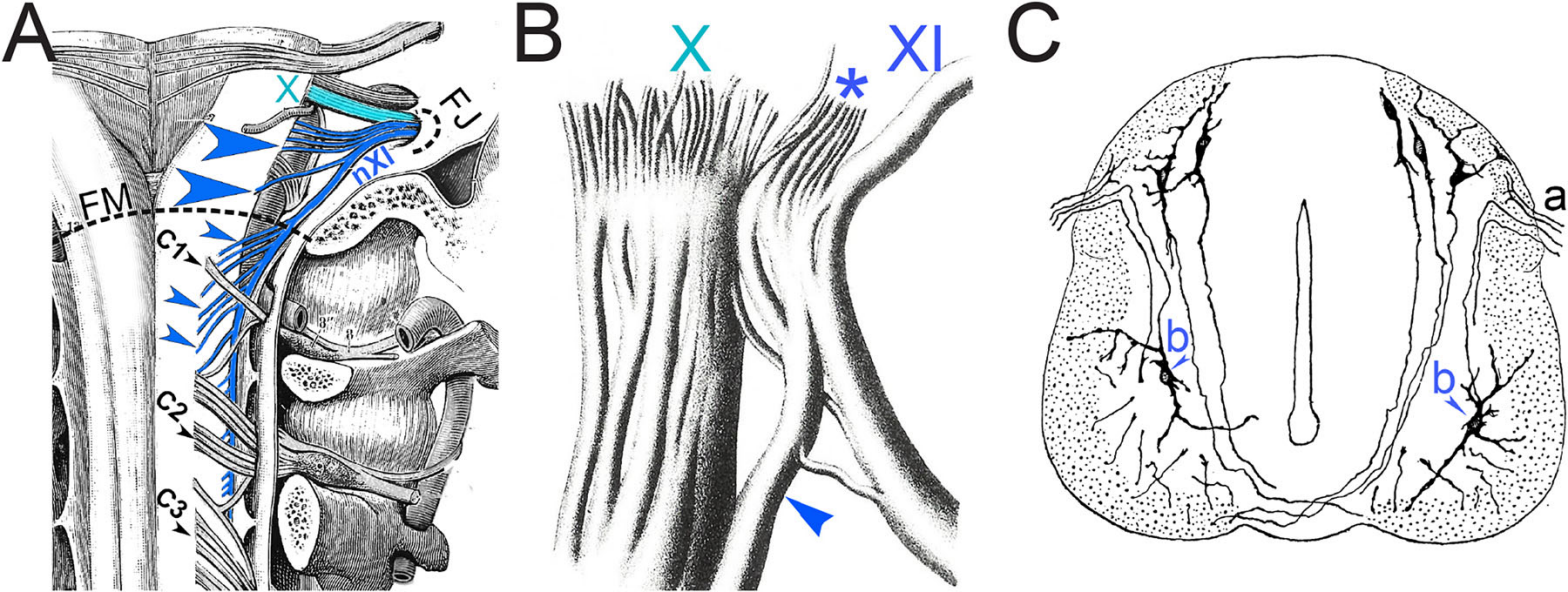
Anatomical configuration of the spinal nerve (nXI). (**A**) Dorsal view of the 11th pair of cranial nerves (nXI) (blue) and dorsal roots of the first spinal nerves (C1–C3) (gray) in humans. Small arrowheads show the spinal roots of nXI (below the foramen magnum) [FM], large arrowheads show its bulbar roots, which nXI will donate to nX [cyan] after traversing the jugular foramen [FJ] (adapted from Latarjet and Testut 1948). (**B**) Example of the junction of nX and nXI at the jugular foramen, in humans. The branch that nXI donates to nX (and that contains the projections from the “cranial roots” of nXI, asterisk) is marked by an arrowhead (reproduced and adapted from Bischoff, E.P.E (1977), English edition of the 1865 orginal). (**C**) Transverse section of a chicken spinal cord at 5 days of incubation: (a) posterior [dorsal] root; (b) “cells of the anterior [ventral] horn projecting axons *en route* to the posterior root” (adapted from Figure 2 in Ramon y Cajal (1890), legend adapted and translated from the French original).

Concerning the nerve itself, one can see the confusion as a protracted hangover from the difficulties in dissecting it, due to anatomical oddities:

- *The nerve (nXI) has an unusual trajectory:* As if it was bent on erasing its spinal origins and acquiring a cranial status, after its emergence from the cervical spinal cord, it ascends to enter the braincase (through the foramen magnum) only to exit it again (through the jugular foramen) (Figure 8A) *en route* to muscles of the neck. Hence, its numbering as a cranial nerve despite the “spinal” of its name and origin.
- Inside the cranium and during its passage through the jugular foramen, it anastomoses with the vagus nerve (nX) (Figure 8B), to the point of forming, according to some authors, a “vagospinal nerve” (Latarjet and Testut 1948 and references therein). Hence the “accessory” of its name: accessory *to the vagus*.
- During its intracranial trajectory but before joining nX, it receives rootlets from the hindbrain (four or five in humans) which have a dorsolateral exit point—typical of branchiomotor nerves—in caudal continuation of the roots of nX. These rootlets have been occasionally dismissed as belonging to nX (Lachman et al. 2002), but extensive documentation of human material leaves little doubt that they are bona fide roots of nXI (Liu et al. 2014; Wiles et al. 2007). Even Van Gehuchten (1908), who deemed these fibers eventually destined to nX, saw them as first joining the spinal root of nXI. No tracing has been done but a constant assumption is that they originate from the caudal part of MoA (sole source of branchiomotor neurons at this level), and are indeed finally destined to nX, more precisely its recurrent laryngeal branch (which can be traced retrogradely to the caudal part of MoA) (Bieger and Hopkins 1987). This conjecture is supported by their recent intraoperative electrophysiological mapping in humans (Brînzeu and Sindou 2017). Székely and Matesz (1993) provides additional arguments from cytoarchitectonics and cytomorphology to classify the origin of the cranial rootlets as belonging to MoA. In conclusion, axons of laryngeal motoneurons originating from MoA briefly course through nXI (conveniently placed nearby), before passing to nX via of a “vagospinal” junction (see above, ii). In and of itself, the transient promiscuity, inside nXI, of motor fibers for the laryngeal muscles with those for the sternocleidomastoid/trapezius cannot decide the nature of the latter (thus of nXI). As it happens, though, it finds a remarkable and satisfying parallel in the fact that both muscle groups are developmentally and even clonally related (Lescroart et al. 2015) (and see below).
- In addition to nXI, the sternocleidomastoid and trapezius muscles receive branches of spinal nerves 1 to 3, as reported over many years, by many authors and in many species (e.g., Edgeworth 1935; Kobayashi et al. 2013; Yan et al.^2007^). Llamas and camels might possess only this cervical innervation (Willemse 1958). This dual innervation suggests either that two types of motoneurons (branchiomotor and somatic) innervate the same muscle—a rare situation, possibly unique in the entire body—or else, that Mo11 is a somatic motor nucleus; however, atypical. The conundrum was finally resolved, at least in chicken: the motoneurons that project to the sternocleidomastoid and trapezius by way of spinal nerves are indistinguishable, in location and genetic makeup (see Table 2) from those that do so via nXI (Kobayashi et al. 2013). Their sole originality is that their axons make a short circuit to the muscle through the roots of C1–C3—as it turns out: their *dorsal* roots, which are thus the only dorsal spinal roots that contain motor axons, an oddity spotted as early as Ramon y Cajal (1890) (Figure 8C). This quite aberrant trajectory might be viewed as a stochastic navigational error permitted by the extreme proximity of the roots of nXI (dorso‐lateral, as befits a branchiomotor nerve) and the dorsal (sensory) roots of cervical nerves (Figure 8A).

In addition to the quirks of the nerve, confusion has been fueled by uncertainties about the nature of the target muscles, the sternocleidomastoid/trapezius (*cucullaris* in fish and birds). Edgeworth (1935) and a few others saw its origin in the branchial “muscle plate”, but a thread of research through the 1990's made them derive from anterior somites. If this were true, the general correspondence between type of motoneuron and type of muscle, mentioned above, would predict that Mo11 is somatic. However, this view was later corrected by some of its very authors, who explained away their previous results by contamination of grafts of somitic mesoderm with nearby non‐somitic mesoderm (Theis et al. 2010 and references therein). Thus, experimental embryology now agrees with lineage tracing (including retrospective clonal analysis), with markers and with genetic dependencies, to establish that the *cucullaris* in birds and sternocleidomastoid/trapezius in mouse do not originate in somites or lateral plate mesoderm, but in a caudal extension of the head (branchial) mesoderm, lateral to the first somites (Heude et al. 2018; Lescroart et al. 2015). Moreover, these muscles obtain (in part) their connective tissue from the neural crest, as befits head muscles (Matsuoka et al. 2005; Theis et al. 2010; Heude et al. 2018).

All in all, it is time to quietly reaffirm the insightful tradition that goes back at least to Edgeworth (1935),—based at the time on fewer data than are now available, and admittedly mixed up with some mistakes, such as the notion that the *cucullaris* does not exist in birds (Edgeworth 1935, p.146)—: the so‐called “spinal accessory nucleus” is a typical branchiomotor nucleus that innervates one or two typical branchiomeric muscles. Let's only dispel the notion that it is “accessory” to anything. It differs from other branchiomotor nuclei only by its caudal position at the ill‐defined hindbrain‐spinal cord boundary, and by some hodological oddities that stem from this location (see above). If the SAN were renamed, it could be called the *spinal nucleus* (SN) giving rise to the *spinal nerve*—that was indeed the French nomenclature in 1920 (Latarjet and Testut 1948). Alternatively, and to preserve coherence with other cranial nerves and nuclei, the nerve can be designated by its other classical name, the 11th pair (nXI), and its motor nucleus called Mo11.

### 3.3 Inner Ear Efferents

The hindbrain harbors a third type of efferents that belongs to the same cardinal class as the first two, but projects to unusual targets, in the inner ear: the outer hair cells of the cochlea and the presynaptic termini of spiral and vestibular ganglionic neurons (Roberts and Meredith 1992). These projections travel in the eighth pair of cranial nerve—which is thus not the purely sensory nerve of most textbooks (e.g., Kandel et al. 2000), but mixed sensory/motor, or at least efferent (but, as we have already seen for preganglionic neurons, developmental genetics justifies the conflation of these terms). If it were not for the striking dispersion of its cell bodies (see below), one could almost add a Mo8 to the list of cranial motor nuclei.

**FIGURE 9:**
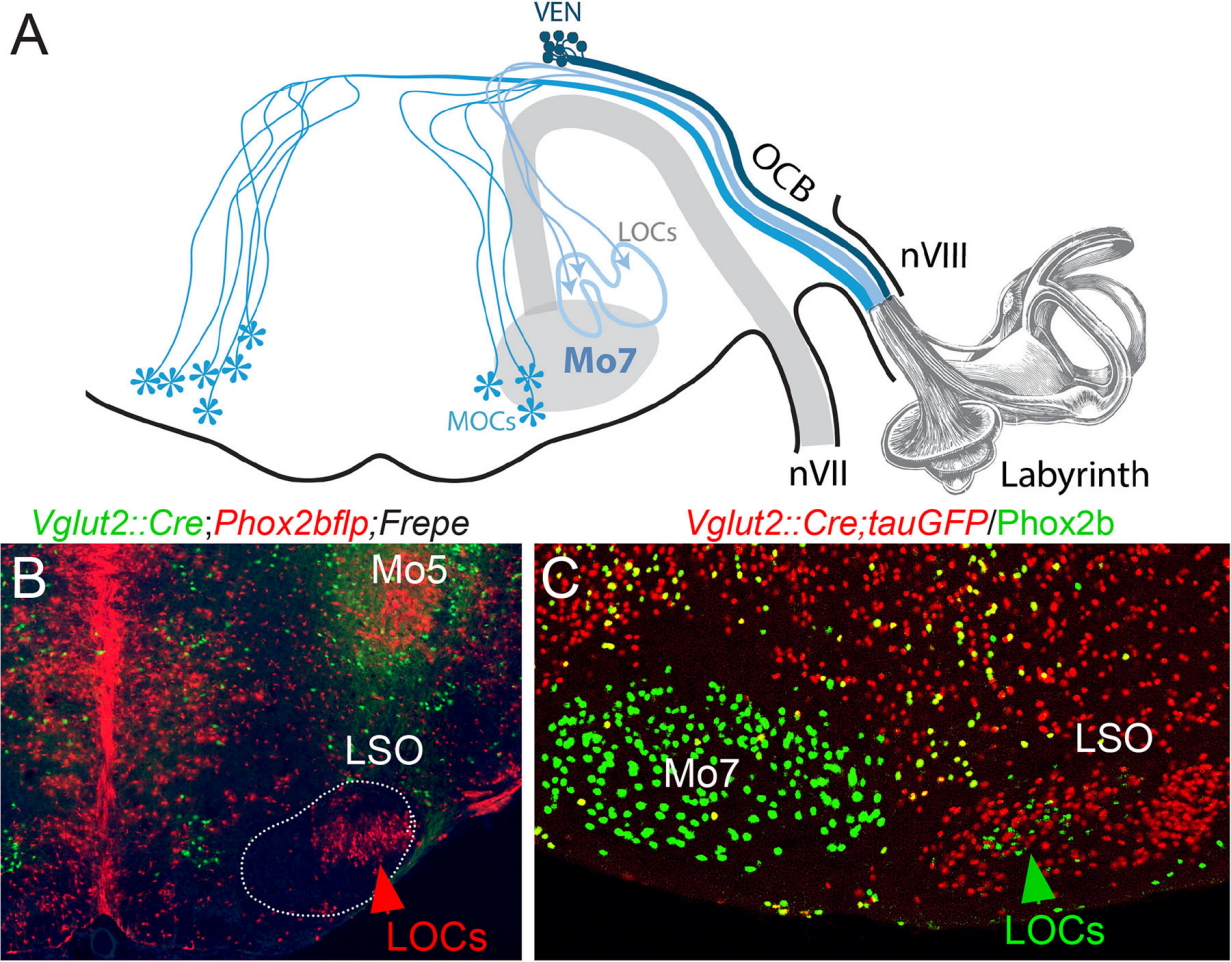
Anatomic configuration of inner ear efferents. (**A**) Semi‐schematic of the three main groups of inner ear efferents, adapted from Brown (2011) and Simmons et al. (2011): the vestibular efferent nucleus (VEN) (often split in two or even three groups, only the so‐called “e‐group” is represented), the medial cochlear efferents (MOCs) (most prominently occupying the ventral nucleus of the trapezoid body), and the lateral ones (LOCs) in the lateral superior olive. The facial nucleus (Mo7) is mostly in another plane of section. Depending on species, each group of cochleovestibular efferents can project bilaterally—only the bilateral contribution of the MOCs is shown. (**B**) Immunodetection at P0 of putative LOCs in mouse (not identified in Kang et al. (2007), possibly because of down‐regulation of *Phox2b* in the adult) on a transverse section of the hindbrain, in a genetic background that labels *Phox2b* ^+^ cells (or their progeny) in red, and the fraction thereof that express *Vglut2* in green (thanks to the RC:FREPE transgene (Bang et al. 2012)): a group of non‐glutamatergic *Phox2b* cells occupies the lateral superior olive, and likely correspond to LOCs (a definitive identification would require tracing from the periphery). (**C**) Immunodetection of *Phox2b* cells on a parasagittal section of the hindbrain at P0 in a genetic background that labels *Vglut2* ^+^ cells in red (thanks to the Tau‐mGFP transgene (Hippenmeyer et al. 2005)): similar to (B), a group of non‐glutamatergic *Phox2b* cells with small nuclei occupies the LSO. LOCs: lateral olivocochlear efferents; LSO: lateral superior olive; Mo5: trigeminal motor nucleus; Mo7: facial motor nucleus; nVII: facial nerve; nVIII: vestibulocochlear nerve (only its efferent fibers are represented); OCB: olivocochlear bundle.

The IEEs are born together with facial branchiomotor neurons in the pMNv neuroepithelial domain of rhombomere 4 (see Di Bonito and Studer 2017; Frank and Goodrich 2018; Fritzsch and Elliott 2017; Simon and Lumsden 1993 for reviews). They part ways with facial motoneuronal precursors (which migrate caudally) to follow an original path, first through the floorplate to the contralateral side (at least for part of them (Simon and Lumsden 1993; Tiveron et al. 2003)), then along a path that soon splits to form the vestibular efferent nucleus (VEN, dorsolateral to the genu of the facial nerve), and the cochlear efferents (CEN, ventrolateral, in the superior olivary complex) (Figure 9A). The CEN neurons project their axons towards the genu of the facial nerve (where they are joined by those of VEN), then laterally to its descending branch to form the “olivocochlear bundle”, and exit the brain through the vestibulo‐acoustic nerve (Fritzsch and Nichols 1993). Their roles, incompletely understood, include protection against acoustic trauma, and modulation of stato‐acoustic afferences (Frank and Goodrich 2018).

These IEEs share with branchiomotor neurons the transcriptional signature *Phox2a*/*Phox2b*/*Tbx20* (see Tiveron et al. (1996, 2003) for the VEN; Kang et al. (2007) for the medial olivocochlear efferent neurons (MOCs) [in the ventral nucleus of the trapezoid body and the ventral nucleus of the lateral lemniscus]; and Figure 9B,C for *Phox2b* in the lateral olivocochlear neurons (LOCs), in and around the lateral superior olive). *Phox2b*‐dependency was examined only for vestibular efferents (Tiveron et al. 2003). Two markers that distinguish IEEs from other branchial efferents are *Gata3* and *Gata2* (see Table 2); the overall genetic proximity of IEEs with branchiomotor neurons suggest a common ancestry (Fritzsch and Elliott 2017).

In conclusion, not only does the pMNv domain of r4 simultaneously give rise to facial motoneurons and vestibular and cochlear efferents, but the latter fall in at least three types by the latest estimate based on transcriptomics (Frank et al. 2023), and possibly more, based on the “puzzling array” of morphological and neurotransmitter phenotypes of the LOCs (Frank and Goodrich 2018) which are suggested, on evolutionary grounds, to be “independently derived” (Roberts and Meredith 1992). The progeny of pMNv in r4 thus offers a striking reminder that mechanisms of neuronal diversification in the central nervous system are yet to be discovered, beyond the spatial and temporal patterning of the neuroepithelium demonstrated in the 1990s.

## 4 Spinal Efferents

The third cardinal class of efferents, like the former two, comprises direct and indirect ones, highly related through their ontogeny: so‐called “somatic” motoneurons and sympathetic preganglionic neurons, located in the spinal cord, with two outliers in the hindbrain. The kinship of all spinal efferents, direct and indirect, is evidenced by their origin in a common progenitor pool (pMNs, adjacent and dorsal to pMNv, “v” standing for ventral, or vagal) as originally inferred from descriptions of their migration pattern (Levi‐Montalcini 1950; Markham and Vaughn 1991), lineage tracing (Leber et al. 1990), and markers (Phelps et al. 1991), and by their common transcriptional signatures and dependencies (summarized in Table 2). This said, differences are to be expected at a subtler level, and indeed direct and indirect motoneurons could be distinguished on the quantitative basis of the averaged expression of 76 homeodomain proteins (Smith et al. 2024).

At the caudal end of this group, in the sacral spinal cord, the indirect efferents (i.e., preganglionics) were originally classified as “parasympathetic” (Langley 1921). The intermediolateral column at that level thus became a “sacral parasympathetic nucleus”. We have provided elsewhere a history and critique of this notion (Brunet 2025; Espinosa‐Medina et al. 2018) and argued that we should forego this spinal instantiation of the so‐called “parasympathetic outflow,” and recognize the sympathetic unity of the spinal autonomic outflow (Brunet 2025) which could become another name for the sympathetic outflow (see also Fritzsch et al. 2017).

This review being devoted to cranial nerves, we restrict our detailed treatment of the spinal class of motoneurons to its cranial outliers, situated in the hindbrain, yet sharing with spinal motoneurons their exit point and cell body position—original reasons for their constant classification as somatic, at least since (Herrick 1918) despite the needless “special” qualification—which reflect a common progenitor domain, and common transcriptional signatures and dependencies (see Table 2): Mo6 (abducens nucleus), and Mo12 (hypoglossal nucleus) and its understudied, and so‐far unnamed continuation in the cervical spinal cord, that projects in cervical nerves, that we name MoC.

### 4.1 Abducens Nucleus (Mo6)

Mo6 innervates the ipsilateral lateral rectus muscle that “abducts” (i.e., pulls on the side) the eyeball. Genetically, it is a bona fide somatic nucleus (i.e., of the type predominantly found in the spinal cord) (see Tables 1 and 2) born from r5, r6 or both rhombomeres, depending on species (Horn and Straka 2021).

Mo6 is thus a puzzling example of evolutionary “bricolage”: it represents a rare—possibly unique—mismatch between type of muscles and type of motoneuron, or, said in another way, a rare case of evolutionary convergence of different neuron types (midbrain/visual and spinal/somatic) on a similar anatomy and physiology (motor neurons for moving the eyeball). It would be interesting to compare the transcriptome of Mo6 with that of Mo3/4 to unravel at what level the convergence occurs in the molecular make‐up of these neurons. The historical and still frequent misattribution of Mo3 and Mo4 to the somatic category (see above) obscures the oddity of this situation, which is a basal feature of extent vertebrates, already present in lampreys (Fritzsch et al. 1990).

How this motor nucleus of the spinal type (one of two in the hindbrain, with Mo12) came to innervate, not a somite‐derived muscle like all the others do (including Mo12), but a non‐somite derived muscle, that belongs in the same class as five other eye muscles innervated by the midbrain Mo3 and Mo4? And how this nucleus came to be recruited in eye movements despite its unwieldy distance from, both, the optic tectum and the other ocular motoneurons? One consequence of this distance is that the coordination, during horizontal eye movements, of the lateral rectus of one eye with the medial rectus of the contralateral eye, relies on a rather acrobatic feat of wiring: a group of interneurons next to Mo6 (in some species intermingled with it) that target the medial rectal pool of contralateral Mo3 (Horn and Straka 2021).

The history of eye muscles and their innervation cannot be retraced from comparative anatomy or paleontology, since all jawed vertebrates possess their whole complement (hagfish has none and the lamprey lacks only a medial rectus (Fritzsch 2024)). One is left to speculate, which we shall unabashedly venture here. On the parsimonious assumption that the mesencephalic Mo3/4 and the rhombencephalic Mo6 did not appear simultaneously, it is tempting to designate Mo6 as the best candidate for the primordial extraocular motor nucleus, on three accounts: (i) its target muscle (the lateral rectus) allows horizontal movements of the eye, likely the cardinal movement, in fish, for “egocentric gaze stabilization,” itself proposed as the original role of ocular mobility: to move the eye relative to the body, but in order to immobilize it relative to the environment during bodily motions (Walls 1962); In animals that have no binocular vision, action of the lateral rectus on one side could conceivably be uncoupled with the action of the medial rectus on the other side; the altogether absence of a medial rectus in lamprey (where the abducens is *de facto* alone in charge of lateral movements), whether it is ancestral or not, is a sign of this dispensability. (ii) Mo6 lies close to the vestibular nuclei which provide the essential input for such a role. (iii) It uses a neuronal differentiation program (for somatic motor neurons) that predates vision (and even the deuterostome/protostome split (Nomaksteinsky et al. 2013)). 0ne can thus propose that a hindbrain outlier of the spinal motor column was recruited to innervate a muscle newly evolved for the eye (the ontogenetic mismatch between muscle and neuron type reflecting this moment of evolutionary “bricolage”). Later, to innervate a more sophisticated eye musculature, a whole new motor complex arose at the isthmus under the control of *Phox2a*—that is after the single *Phox2* gene of the vertebrate ancestor, in charge of branchial motoneurons (Dufour et al. 2006), was duplicated into *Phox2b* (which kept this ancestral role), and *Phox2a*, left free to acquire novel roles in novel territories: Mo3/Mo4, but also the epibranchial placodes (Dauger et al. 2003) and the locus coeruleus (Morin et al. 1997; Pattyn et al. 2000). Altogether, it seems more intuitive to imagine that Mo6 persisted as a relic of an earlier, simpler age of ocular motricity, than to imagine the late recruitment of this distantly located somatic nucleus to take charge of the only extraocular muscle (out of six) that, somehow, Mo3/4 could not innervate despite being impeccably designed and conveniently placed for that purpose. The condition in lamprey (if ancestral) where Mo6 innervates two muscles instead of one (Fritzsch 2024)—thus presumably controls a slightly more elaborate pattern of eye movements—could be another trace of this scenario.

On a practical note, all motoneurons for eye muscles are largely resistant to amyotrophic lateral sclerosis and spinal muscular atrophy (Kanning et al. 2010), including those in Mo6 (Ferrucci et al. 2010), which are of the same cardinal class as the sensitive motoneurons of the spinal cord. The correlation of resistance with the muscles innervated rather than the motoneuron class could suggest a non‐cell autonomous causality rather than the class‐specific cell‐intrinsic factors actively sought in Mo3 or Mo4 (e.g., Allodi et al. 2019; An et al. 2019; H. Lee et al. 2021).

### 4.2 Hypoglossal Nucleus (Mo12) and the Unnamed Nucleus for the Infrahyoid Muscles (MoC)

The hyoid bone, free floating relative to other bones, is the site of attachment of three classes of muscles which connect it, either to more rostral bones (the “suprahyoid” muscles), or to more caudal ones (the “infrahyoid” muscles), or which protrude inside a mucosal sack (the tongue muscles). Suprahyoid muscles are branchiomeric and innervated accordingly by the branchiomotor MoAcc or Acc5/7 (see Part 3.1); while “hypobranchial” muscles (omo‐hyoid, sterno‐hyoid, sterno‐thyroid, and thyro‐hyoid) and tongue muscles are somite‐derived (Adachi et al. 2018 for references) and innervated accordingly by somatic motoneurons, which form the hypoglossal nucleus (Mo12) for the tongue and, for the infrahyoids, a ventral column in the upper cervical spinal cord. The latter, although cytoarchitecturally distinct (Gottschall et al. 1980), has yet, for some reason, to reach the status of “nucleus” in the anatomic literature. We hereby correct this situation by proposing that they constitute an “infrahyoid nucleus,” abbreviated as MoC on account of its projection in the cervical spinal nerves C1, C2, and C3.

**FIGURE 10:**
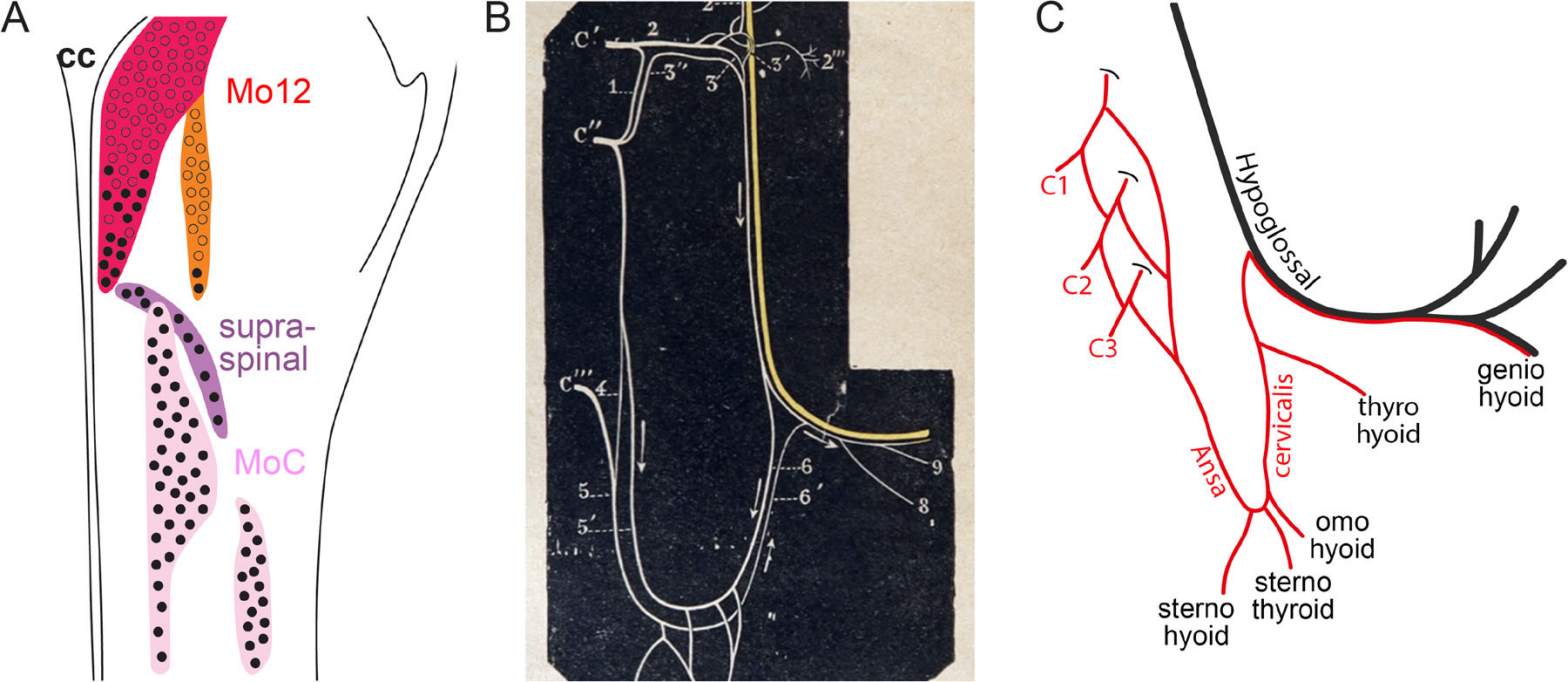
Motor nuclei and nerves for the infrahyoid and tongue muscles. (**A**) Outline of a hemi‐caudal medulla oblongata of rat, with location of motoneurons for the tongue and hypobranchial muscles (adapted from Kitamura et al. (1986)). Motoneurons are color‐coded red (tongue), orange (geniohyoid), purple (thyrohyoid), and pink (other infrahyoids). Empty dots are neurons projecting through nXII, filled dots are neurons projecting through cervical nerves. Cc: central canal. (**B**) Semi‐schematic representation of the hypoglossal (yellow) and first three cervical nerves (C′, C″, and C‴, white) according to Latarjet and Testut (1948). The cervical nerves join nXII through both its main trunk and *ansa cervicalis* where both contributions meet (arrowheads). (**C**) Same structures reproduced with permission from Kitamura et al. (1986), whereby the first three cervical nerves join nXII exclusively through the *ansa cervicalis*. The geniohyoid is mostly innervated by a portion of Mo12: this justifies that it might be best viewed as a fascicle of the hyoglossus tongue muscle (Latarjet and Testut 1948) despite its suprahyoid status in most accounts. Its derivation from somites, unexplored to our knowledge, would settle the case.

The kinship of Mo12 and MoC—as “expected from the phylogenetic and ontogenetic relationship of [their target] muscles” (Kitamura et al. 1986)—which justifies the inclusion of MoC in a review on cranial nerves, is manifested centrally by a thin bridge between them that constitutes a “supraspinal nucleus” (Figure 10A) (Kitamura et al. 1986); and also in the periphery by a spectacular loop that the axons of MoC execute to join nXII, called the *ansa cervicalis* (Figure 10B,C). This *ansa* abandons several branches (through which MoC projects to the hypobranchial muscles) before joining nXII to contribute a modicum of fibers to the tongue and geniohyoid (otherwise mostly innervated by Mo12) (Figure 10B,C).

## 5 Conclusion: A New Classification for All Efferents

An ontogenetic (and by inference evolutionary) perspective on efferent neurons of the central nervous system encourages to group motor neurons in the strict sense together with preganglionic neurons of the autonomic nervous system. Even before genes were a thing, Langley had found a “fundamental resemblance between […] all the efferent fibers which leave the spinal cord whether they end in multi‐nuclear striated muscle or in peripheral nerve cells” (Langley and Anderson 1904), based on a developmental observation: they readily exchanged post‐synaptic partners upon surgical rerouting. By the same type of experiment (Wigston and Sanes 1985) later showed that preganglionics even maintained their capacity to detect rostro‐caudal addresses on an illegitimate muscle target. Our new classification highlights that this resemblance is paralleled by—and likely rooted in—genetic signatures. Assuming that indirect effectors and their neural crest‐derived ganglionic targets are evolutionarily younger than their direct counterpart, their kinship at three levels of the neuraxis suggests that the former were derived from the latter three times in the vertebrate lineage. For example, the midbrain indirect pathway, apparently lacking in lampreys together with intrinsic muscles of the eye (Nilsson 2011; Pombal and Megías 2019 and references therein), could have arisen—if the condition is primitive—after the split between cyclostomes and gnathostomes.

Thus, the age‐old designation of preganglionic neurons as “visceral motor neurons” is fully justified (and their role is motor in the strict sense whenever their target, albeit indirect, is muscular). The only amendment to the terminology should be to drop the “general” and “special” terms used at the level of the hindbrain (as also advocated by Fritzsch et al. 2017) for, respectively, indirect (i.e., preganglionic) and direct (i.e., branchial) efferents: there is nothing “general” about indirect efferents of the hindbrain, which are just as “special” to this rostro‐caudal level as their direct counterparts.

On the other hand, and less intuitively, the same ontogenetic perspective leads to radically distinguish three classes of motor neurons (each comprising a direct and an indirect subclass) that occupy different rostro‐caudal levels in the CNS: midbrain, hindbrain and spinal cord (notwithstanding the marginal anatomical overlap of the latter two classes). The depth of this ontogenetic separation is such that the category “motoneuron” cannot be considered as reflecting a cell type, even in the broadest sense, and appears as vague as that of interneuron.

It remains that all three classes of motor neurons, direct and indirect, share some fundamental features: they project out of the CNS (their very definition) even if it is through different types of roots, they synapse onto muscles or ganglionic neurons, and they are cholinergic (their least specific feature). Three distinct ontogenies could thus be said to “converge” onto these common features. It would be interesting to figure out whether these common features are underlaid by common genes (aside from the trivial case of cholinergic neurotransmission), and if so, at what level of the neuron's differentiation trajectory distinct upstream regulators converge onto common downstream effectors.

From a developmental standpoint, our classification reconciles, in a gratifying but almost trivial way, cell types and body plan: no more somatic motoneurons lost in the midbrain, no more parasympathetic ones sneaking into the sacral spinal cord, but a registration of neuron types with the rostro‐caudal axis of the central nervous system—and, among other things, its *Hox* code. The old model entailed that different regions of the central nervous system would produce the same cell types, introducing a mystifying discontinuity between early and late phases of embryonic development. We are not aware that a similar claim was ever made for other neuron types.

Physiology, like anatomy, is the endpoint of development. Strikingly, the new model suggests a matching between developmentally‐defined cell types and the most integrative level of physiology conceivable (despite discordant details to which much of this review is devoted). In the spirit of Romer (1972) who, based on anatomy, physiology, and development (albeit not genetic at the time), held that the vertebrate animal was “dual, visceral, and somatic,” we propose, rather, that it is triple: (i) *Visual*, defined by all midbrain efferents, direct and indirect, and all eye muscles. (ii) *Vegetative*—in a throwback to Bichat (1802)—or *anabolic*—referring to Gaskell (1920)—or *interofective*—in a repurposing of Cannon's terminology (Cannon 1939)—defined by most hindbrain efferents, direct and indirect, and their targets, primarily involved in feeding—from its oral to its intestinal phases—, breathing—ancestrally through gills—, and attending slowing of the heart; and (iii) *animal* (Bichat 1802), *catabolic* (Gaskell 1920) or *exterofective* (Cannon 1939) comprising direct spinal efferents and their targets (trunk and limb muscles), but also, in a crucial modification of the original terms, the indirect (sympathetic) spinal efferents, also largely devoted to the mobilization of the body in the environment by attending to its cardiovascular requirements: vasoconstriction and heart stimulation, for “fight or flight,” to which one might deem too risqué to add a third “f” word to include sexual activity, also sympathetic (Espinosa‐Medina et al. 2016; Sivori et al. 2024; Brunet 2025). Even the hypoglossal nerve, a cranial outlier of the spinal series, fits in this general registration if one considers that in many species the tongue (derived from somites like locomotory muscles) acts as a prehensile organ that brings external objects into the mouth (water in species that lap, food in many amphibians and reptiles), that is as a sort of fifth limb.

**FIGURE 11:**
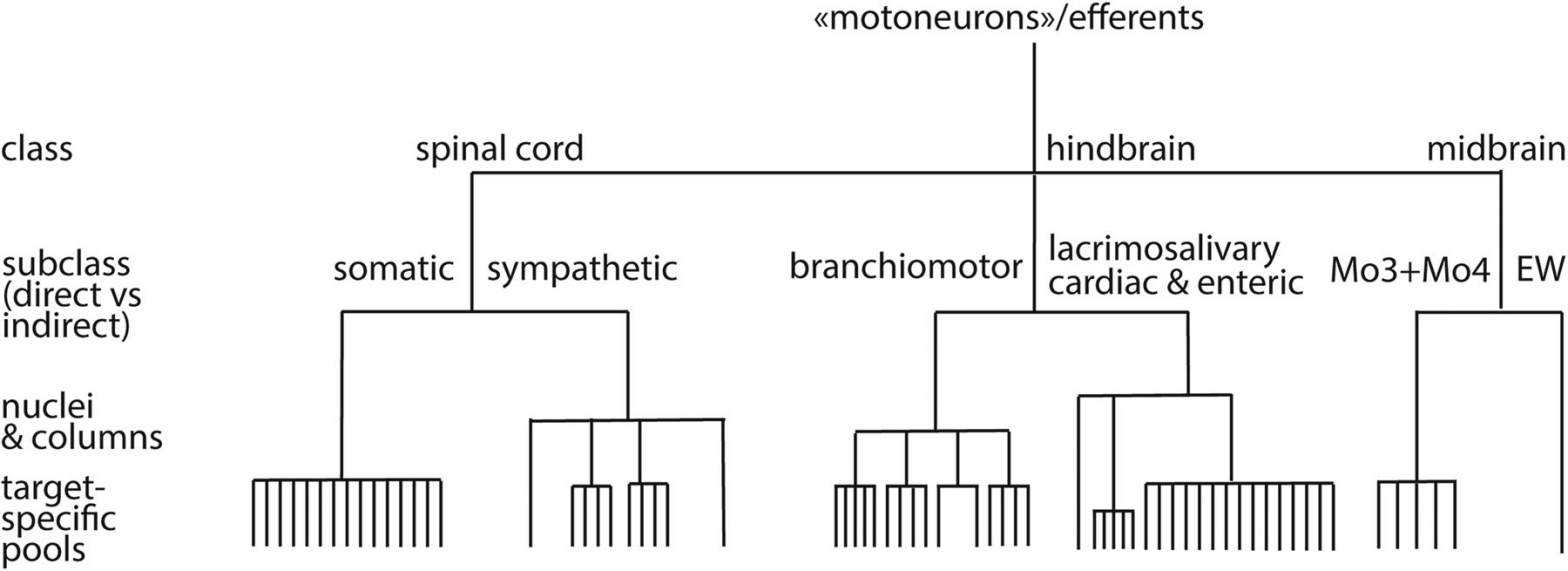
Prototype of a cladogram of all efferents based on available genetic evidence. All efferents are placed on a cladogram that takes into account genetic evidence, but not the distances of the transcriptomes, which are yet to be measured directly. No data so far allows to infer the distance between midbrain, spinal, and hindbrain efferents, hence their polytomic arrangement on the cladogram. Subtypes of neurons are documented for the sympathetic (see Part 1), somatic (Alkaslasi et al. 2021; Blum et al. 2021), and branchiomotor (e.g., Tenney et al. 2019) categories, and are likely in lacrimo–salivary from the UMAP of the sphenopalatine in Sivori et al. (2024) and expected in Mo3/Mo4, based on the existence of subnuclei for individual muscles (Büttner‐Ennever 2006). In the schematic, the number of the lower rung subdivisions is arbitrary. Such a cladogram should not be read as a phylogenic tree (Zeng and Sanes 2017) although it suggests that different classes arose at different nodes of such a tree.

The potentially jarring aspect of this grouping is that it cuts across the time‐honored and deeply engrained notion of *visceral* or *autonomic* outflow, by fracturing it into three components and highlighting instead the affiliation of each component to a corresponding set of direct efferents (Figure 11). But this new classification is true to both, the paramount roles of each of these three components and their distinct ontogenies (and by implication, evolution). The visual component is detached from the former “parasympathetic” (with which it always formed an awkward assemblage, having nothing to do with homeostasis to begin with); and the sympathetic, including the pelvo‐sympathetic (Sivori et al. 2024; Brunet 2025), in other words the spinal outflow, is integrated in the former *somatic* animal of Romer, *animal* of Bichat and *exterofective* of Cannon, in line with its principal role in enabling actions in the external world, and adaptations to it: excitatory innervation of the heart, arterial tree, piloerector muscles, and sweat glands (the latter three distributed throughout the muscle mass and teguments and representing problematic “viscera” to begin with), glucagon secretion by pancreatic α‐cells, and thermogenesis and lipolysis in adipose tissue (Martinez‐Sanchez et al. 2022 for review). Even pelvic functions (defecation, micturition, and copulation), with their intertwined voluntary and involuntary control, are more easily understood as engagements with the environment than purveyors of homeostasis.

The partition of the autonomic outflow in three systems also helps recognize the extent to which they control non‐overlapping domains, and to which many of the overlaps appear like evolutionary afterthoughts, secondary invasions of the domain of one system by another. The spinal outflow has a considerable reserved domain: most of the arterial tree, piloerector muscles, and sweat glands, fat tissue, bladder, sexual organs, rectum. The midbrain outflow also: accommodation of the eye to light or distance is entirely under its control, the spinal outflow providing only a tonus to the dilator of the iris (Clarke et al. 2003; Lowenstein 1950), the functional equivalent of a more elastic iris. The hindbrain outflow is proportionally more conducive to partnership (with the spinal outflow, most obviously on the heart), yet provides the only preganglionic neurons to the enteric nervous system (which is impinged on by *ganglionic* cells of the spinal outflow, an unusual three‐neuron arrangement, see in Jänig 2006, p.203), as well as the main input to salivary glands (the spinal outflow being confined to a marginal role, synergistic with, and modulatory of the hindbrain one—its anti‐salivary action, already denied by Langley (1878), a “myth […] that has resisted proper burial” (Garrett 1987)). It is only to be expected that evolution, playing with the vast array of autonomic projections, has tinkered, for better physiological integration, occasional promiscuities and interplays—which are the legitimate focus of much contemporary research—but they only blur, not to the point of obliteration, the contours of a largely partitioned landscape, presumably ancestral.

## Funding

The study was supported by Inserm (J.‐F.B, M.S.), CNRS (J.‐F.B, M.S.), and ANR‐24‐CE13‐2279‐02 (J.‐F.B).

## Conflicts of Interest

The authors declare no conflicts of interest.

## Data Availability

The raw data in Figures 7 and 9 are available from the corresponding author upon reasonable request.

## Endnotes

1 An exception, so far, might be the expression of the estrogen‐related receptor *Err3*—a marker (Friese et al. 2009) and determinant (Khan et al. 2022) of spinal γ‐motoneurons—in a hindbrain nucleus, Mo5 (Morita‐Isogai et al. 2017), which innervates the only branchial muscles endowed with muscle spindles (Rokx et al. 1985 and references within)—with the caveats that the *Err3*‐expressing neurons in Mo5 were not directly identified as γ, and that in the oculomotor nucleus (Mo3), which innervates spindle‐less muscles in rodents (Maier et al. 1974), most motoneurons express *Err3* (Khan et al. 2022).

2 Which for example inspired the description of these unrelated cell groups as a single entity: ″The Edinger‐Westphal nucleus (EW) is a midbrain nucleus composed of a preganglionic, cholinergic subpopulation, and a densely clustered peptidergic subpopulation (EWcp) (Medrano et al. 2024).

3 So that Reiner et al. (1983) could write: “The location of EW [in mammals] has become particularly unclear.”
